# Motor Adaptation Scaled by the Difficulty of a Secondary Cognitive Task

**DOI:** 10.1371/journal.pone.0002485

**Published:** 2008-06-18

**Authors:** Jordan A. Taylor, Kurt A. Thoroughman

**Affiliations:** Department of Biomedical Engineering, Washington University in Saint Louis, Saint Louis, Missouri, United States of America; Harvard Medical School, United States of America

## Abstract

**Background:**

Motor learning requires evaluating performance in previous movements and modifying future movements. The executive system, generally involved in planning and decision-making, could monitor and modify behavior in response to changes in task difficulty or performance. Here we aim to identify the quantitative cognitive contribution to responsive and adaptive control to identify possible overlap between cognitive and motor processes.

**Methodology/Principal Findings:**

We developed a dual-task experiment that varied the trial-by-trial difficulty of a secondary cognitive task while participants performed a motor adaptation task. Subjects performed a difficulty-graded semantic categorization task while making reaching movements that were occasionally subjected to force perturbations. We find that motor adaptation was specifically impaired on the most difficult to categorize trials.

**Conclusions/Significance:**

We suggest that the degree of decision-level difficulty of a particular categorization differentially burdens the executive system and subsequently results in a proportional degradation of adaptation. Our results suggest a specific quantitative contribution of executive control in motor adaptation.

## Introduction

Monitoring performance and updating future behavior is an essential process underlying motor adaptation. Numerous studies have identified key transformations that map sensory experience to future motor behavior and have developed theoretical models to describe these processes [1]–[4]. A tacit assumption common to this body of work is that motor adaptation is largely automatic. However, the process of monitoring performance and modulating future behavior in cognitive tasks, such as the Stroop task or Flanker task, has been assigned to the executive system [5]–[7]. The executive system, which is commonly referred to as attention or cognitive control, is functionally defined as the mechanism to orient and enhance sensory systems, and to coordinate output systems in a goal directed manner [6]. Orienting sensory systems to behaviorally relevant environmental stimuli [8] and enhancing information processing [9], [10] is commonly referred to as attention, while defining goals and coordinating behavior is commonly referred to as cognitive control [5], [6]. The executive system encompasses both of these processes to guide future behavior and monitor ongoing performance. Both successful motor adaptation and accurate performance on cognitive tasks require performance monitoring and updating behavior following errors; therefore, these disparate tasks may share overlapping processing. We ask here if the executive system plays a significant role in motor learning.

Several previous studies have utilized dual-task manipulations to interrogate the role of the executive system in motor learning and to determine the degree of automaticity of motor skills. Most of these studies have focused on motor sequence learning by using a serial reaction time task combined with a secondary task. Participants do not implicitly nor explicitly learn the sequence when distracted by a secondary task [11]–[13]. While these studies have established dual-task interference effects in sequence learning, sensorimotor adaptation has been suggested to be functionally and neurally distinct from sequence learning [14].

The effect of divided attention on sensorimotor adaptation has only been briefly investigated. Sensorimotor adaptation [15]–[17] and motor skill learning [18] are impaired when participants' attention is divided between a motor task and a secondary cognitive task. However, these studies have employed a variety of secondary tasks, ranging from tone counting to mental arithmetic; therefore, the relationship of secondary task burden and resultant motor impairment is difficult to ascertain. If the secondary task utilizes a process that is shared between the motor task and secondary task, then the processing demand of the secondary task should proportionally impair the motor task.

In a previous study from our lab [17], we attempted to determine the relationship between secondary task difficulty and motor adaptation. In a dual-task motor adaptation study, participants performed reaching movements that were occasionally perturbed by a robotic manipulandum. In addition, on each movement participants performed an auditory frequency discrimination task (FD), in which they judged the change in pitch between two sequentially presented tones. The timing between tones and the difficulty to discriminate the tones was varied. We found that when the FD task was temporally coincident with a movement error in the perturbed movement, then adaptation on the following movement was significantly impaired. However, the difficulty of the FD task did not affect motor adaptation. This indicates that the impairment in adaptation that we observed may only be due to a timing or distraction effect from the FD task rather than a dual-task burden on the executive system. Therefore, the contribution of executive systems to motor learning may only be to direct attention to movement and may not play an active role in motor adaptation. Alternatively, it is possible that the secondary task was not difficult and did not burden the executive system.

The secondary task we employed was data limited; perhaps a secondary task that is decision limited could be better suited to examine the role of the executive system in motor learning. In data-limited tasks, performance is determined by the quality or structure of the input data; expending additional effort does not improve performance on data-limited tasks [10]. In decision-limited tasks, performance is determined by the amount of effort exerted on the task. Decision-limited tasks, such as semantic or perceptual categorization tasks, provide some quantification of the burden of the task by measuring subjects' reaction time for each categorization [19]–[21]. The more difficult an item is to categorize, then the longer the reaction time is for that categorization [22]–[24]. The increased reaction time provides an indication of the processing demands placed on the system by the categorization task on that trial and the resultant degradation of the primary task can be correlated. By utilizing a decision-limited task to burden the executive system during motor learning, we can determine if the executive system plays an active role in motor adaptation.

We designed an experiment to specifically burden the executive system by having subjects perform a semantic categorization task while performing a motor adaptation task. Utilizing a semantic categorization task allowed the secondary task to vary in decision-level difficulty trial-by-trial. Subjects performed horizontal reaching movements, which were occasionally subjected to a transient force perturbation. On some movements, subjects made concrete or abstract word categorizations, on an aurally presented word, by pressing a corresponding button with their left hand. Some words fit well into concrete or abstract categories, while some of the words were ambiguous to which category best labeled the word. The categorization task difficulty differentially influenced subjects' reaction times and adaptation on a trial-by-trial basis. Within-movement feedback control was not affected by the categorization task, but across-movement adaptation was impaired. Subjects were the slowest to categorize ambiguous words and adapted the least following trials in which an ambiguous word was presented as compared to trials in which a concrete or abstract word was presented. This interference was specifically related to the degree of category uncertainty of a particular word. These results suggest that the executive system significantly interacts with the motor learning process, graded by the burden on the executive system.

## Results

### Word Semantic Categorization

Words were pseudorandomly presented on the movement before a pulse (prepulse movement), during a pulse movement (pulsed movement), and immediately following a pulsed movement (postpulse movement). On movements without a pulse, subjects were slower to categorize ambiguous words, as measured by subjects' reaction time (RT), compared to concrete words (paired t-test, p = 0.002) and abstract words (paired t-test, p = 0.004) during the dual-task experiment. Nonpulsed RTs to concrete, abstract, and ambiguous words were 1.196±0.210 s, 1.314±0.226 s, and 1.442±0.256 s, respectively. Subjects' RTs to abstract words were, on average, slower than concrete words, but this difference did not reach significance (paired t-test, p = 0.065). Word categorizations on pulsed movements were slower than categorizations on nonpulsed movements (paired t-test, p = 0.039). Pulsed RTs were 1.266±0.212 s, 1.398±0.232 s, and 1.580±0.288 s, for concrete, abstract, and ambiguous word categories. The increase in reaction time from a categorization on a nonpulsed movement to a pulsed movement was 0.070 s, 0.085 s, and 0.138 s, for concrete, abstract, and ambiguous words. Ambiguous word categorizations on pulsed movements are nearly 140 ms longer than ambiguous word categorizations on nonpulsed movements, while concrete categorizations on pulsed movements are increased only 70 ms from concrete word categorizations on nonpulsed movement. This increase in RT is nearly twice as large for ambiguous words as for concrete words indicating that the pulse itself does cause a differential interference on the categorization, however, this trend did not reach significance (p>0.1).

### Prepulse Movements

**Figure 1:**
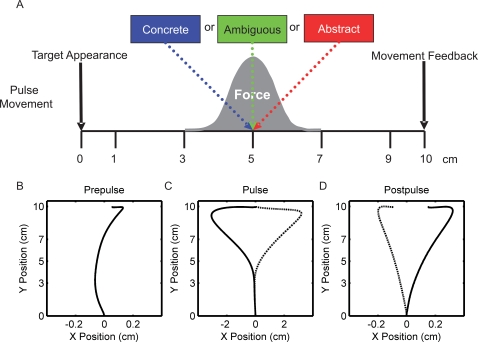
The experimental setup and the averaged movement trajectories for prepulse, pulse and postpulse movements. A) Dual-task setup during pulsed movements. Hand position in the y-direction (toward the target) triggered perturbation force in the x-direction and/or the onset of the word presentation. The force perturbation was centered at y = 5 cm. When the hand arrived at y = 5 cm, either a concrete (blue), abstract (red), or ambiguous (green) word was presented. The word was 500 ms in duration. At the end of movement, subjects received feedback on the correctness of the movement, but no feedback on the categorization was provided. B) Average, across all dual-task conditions, prepulse movement trajectory. C) Average pulse movement trajectories for leftward (solid) and rightward (dashed) force perturbations. D) Average postpulse movement trajectories, minus the average prepulse movement trajectory, following leftward (solid) and rightward (dashed) force perturbation.

Prepulse movements followed approximately a straight-line from the starting position to the target position (figure 1b). Movement curvature, as measured by movement area, varied closely around zero. For concrete, abstract, and ambiguous words, the signed area swept out during the entire movement was 0.151±0.734 cm^2^, −0.070±0.556 cm^2^, −0.255±0.598 cm^2^, respectively. There was no significant difference in curvature between prepulse movements for concrete, abstract, or ambiguous words (3-way ANOVA, p = 0.677). The prepulse perpendicular displacement (PD) at 5 cm into movement was 0.003±0.092 cm, −0.057±0.074 cm, and −0.039±0.077 cm and were not different across concrete, abstract, and ambiguous word presentations (3-way ANOVA, p = 0.578).

### Pulse Movements

**Figure 2:**
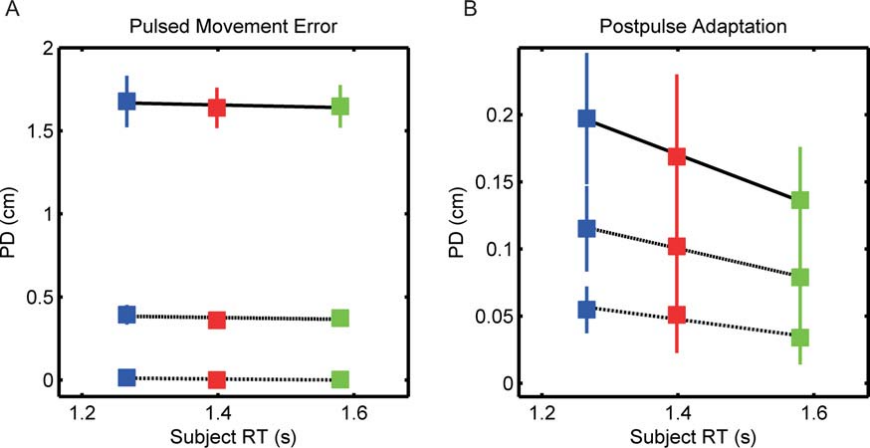
The effect of word categorization reaction time (RT) on within-movement feedback control and across-movement adaptation. Perpendicular displacements at 3 (dotted), 5 (dashed), and 7 cm (solid) in the pulsed (A) and postpulse movements (B) versus subject categorization RT for concrete (blue), abstract (red), and ambiguous (green) word categories in the pulsed movement. A) Word categorization RT was scaled by the semantic category; subjects responded progressively faster depending on the concreteness of the word. Neither semantic category nor RT affected within movement feedback control on pulsed movements. B) Postpulse adaptation was scaled by the word categorization RT. Subjects showed significantly more adaptation following pulsed movements with concrete words than pulsed movements with ambiguous words. Error bars represent the 95% confidence interval of the mean.

On pulsed movements, subjects' hand trajectories were perturbed in the direction of the force pulse (figure 1c). Forces were pseudorandomly presented either to the left or to the right. The peak of the force did not differ across word categories (3-way ANOVA, p = 0.994). The peak force was 12.117±0.476 N, 12.089±0.486 N, and 12.079±0.475 N on pulsed movements in which a concrete, abstract, or ambiguous word was presented, respectively. The duration of the force was not different across word categories (3-way ANOVA, p = 0.477); the averaged force durations were 0.153±0.006 s, 0.148±0.019 s, and 0.160±0.013 s for concrete, abstract, and ambiguous words, respectively. The category of word did not affect the hand displacement on pulsed movements (figure 2a; 3-way ANOVA, p = 0.946). The maximum PD for concrete, abstract, and ambiguous words was 3.300±0.322 cm, 3.365±0.302 cm, 3.367±0.318 respectively. The initiation of the corrective response (Tc), as measured by the time from maximum force until the x-component of the acceleration of the hand changed sign, was not different across word category types (3-way ANOVA, p = 0.796); Tc was 0.052±0.003 s, 0.053±0.002 s, and 0.053±0.002 s for concrete, abstract, and ambiguous words, respectively. To quantify the effect of the word late into movement, we measured the subjects' settling time and integral squared error (ISE) of the movement. The settling time, which was defined as the time from the maximum force until the subject's hand was within 10% of its final value, was not different across word categories (3-way ANOVA, p = 0.963). It took 1.098±0.205 s, 1.070±0.166 s, and 1.104±0.177 s to reach steady state on pulses in which concrete, abstract, and ambiguous words were presented, respectively. In addition, the presentation of the different word categories did not affect ISE (3-way ANOVA, p = 0.993). The ISE was 23.697±4.323 cm^3^, 23.354±3.899 cm^3^, and 23.611±3.971 cm^3^. None of movement metrics revealed a significant difference in movement trajectories between individual word categories using paired t-tests.

### Postpulse Movements

Immediately following the force perturbation, subjects' movements were angled to the right following leftward perturbations (figure 1d – solid) and to the left following rightward perturbations (figure 1d – dashed). Three metrics were used to quantify postpulse adaptation following pulsed movements in which a word was presented, initial movement direction, PD at 3, 5, and 7 cm into movement, and total movement area. Initial movement direction was used to measure adaptation early into movement. The direction angle for concrete, abstract, and ambiguous words was 1.335±0.372°, 1.189±0.527°, and 0.913±0.327°, respectively. The direction angle following concrete words was not significantly different from adaptation following abstract words (p = 0.5561). The postpulse direction angle following ambiguous words tended to be less than adaptation following concrete words (p = 0.084), but not significantly less following abstract words (p = 0.316). Adaptation was more evident later in the movement, as measured by PDs midway into the movement (figure 2b). PDs at 3, 5, and 7 cm were significantly different between word categories (ANOVA with word categories as within-subject factors and PDs at 3, 5, and 7 cm as within-subject factor levels, p = 0.044). Comparison of adaptation following pulses with concrete and abstract words did not reveal significant differences in postpulse movements PD (ANOVA with concrete and abstract as within-subject factors and PD at 3, 5, 7 cm as within-subject factor levels, p = 0.426). For example, PD at 5 cm was 0.114±0.032 following pulses with concrete words and 0.102±0.046 cm following abstract words (figure 2b). However, PD following ambiguous words was 0.079±0.029 cm, significantly less than the adaptation following concrete words (ANOVA with concrete and ambiguous word categories as within-subject factors and PDs at 3, 5, and 7 as within-subject factor levels, p = 0.005). While on average the PD following abstract words was greater than PD following ambiguous words, they were not significantly different (ANOVA with abstract and ambiguous word categories as within-subject factors and PDs at 3, 5, and 7 as within-subject factor levels, p = 0.126). Total movement area, measured from the start to the end of movement, following ambiguous word presentation decreased by 50% (0.876±0.270 cm^2^) from the movement area following concrete words (1.315±0.328 cm^2^; paired t-test between concrete and ambiguous, p = 0.016).

### Offline Word Survey Analysis

**Figure 3:**
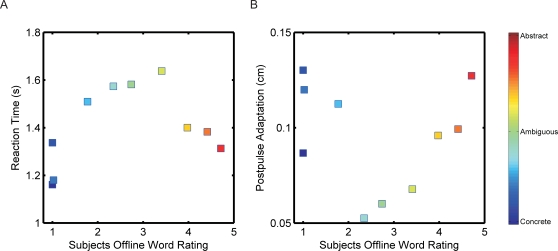
The dependence of categorization speed and adaptation on subjects' semantic conception of words. Offline, subjects categorized words presented during the movement task on a scale of 1 to 5, with 1 being most concrete, and 5 being most abstract. Ratings were separated into 10 equally sized bins and averaged across subjects. Colorbar (side) represents the linear spectrum of subjects' offline word survey of concreteness from most concrete (blue) to most abstract (red). A) Online word categorization RT was the fastest for words that were best categorized into concrete or abstract words offline. Subjects were slowest for ambiguous words, which were words that subjects classified as not falling directly into concrete or abstract categories during the offline survey. B) Subjects adapted the most to words that fell best into concrete or abstract categories, while words that were ambiguous had the least adaptation.

Following the dual task, subjects were provided with a survey of the 150 words they heard during the experiment. The subjects were asked to rate each of the words from 1 to 5 with 1 being concrete and 5 being abstract. This allowed us to probe their categorization of each word with higher resolution and without the distraction of the movement task. The offline word ratings were used to bin and average subjects' online RTs and postpulse PDs. Reaction times for words that were near the ends of the categorization spectrum (ratings of 1 or 5) were faster than words that fell in the middle of the spectrum (figure 3a). Correspondingly, adaptation was the most for words that were near the ends of the categorization spectrum and the least for words that fell in the middle (figure 3b). The relationship between RT and postpulse PD was significantly correlated (slope different from zero, r = −0.642, p = 0.045). Postpulse PD was not significantly correlated with elapsed time between movements (slope different from zero, r = 0.1256, p = 0.729), the HAL frequency of the words (slope different from zero, r = 0.1717, p = 0.635), or lexical decision RT (slope different from zero, r = 0.316, p = 0.3738).

### Comparison of Online and Offline Word Categorization

The subjects had the opportunity to categorize each word online during the dual task and offline during the word survey. Regardless of the word category, subjects maintained their categorization from online to offline on 75.3% of the words. When participants changed their categorization, their average online reaction time was 1.564±0.299 s, which was increased from an average reaction time of 1.399±0.252 s for a maintained categorization. The difference between these reaction times was marginally significant (p = 0.045) when we grouped all categorization changes together.

**Figure 4:**
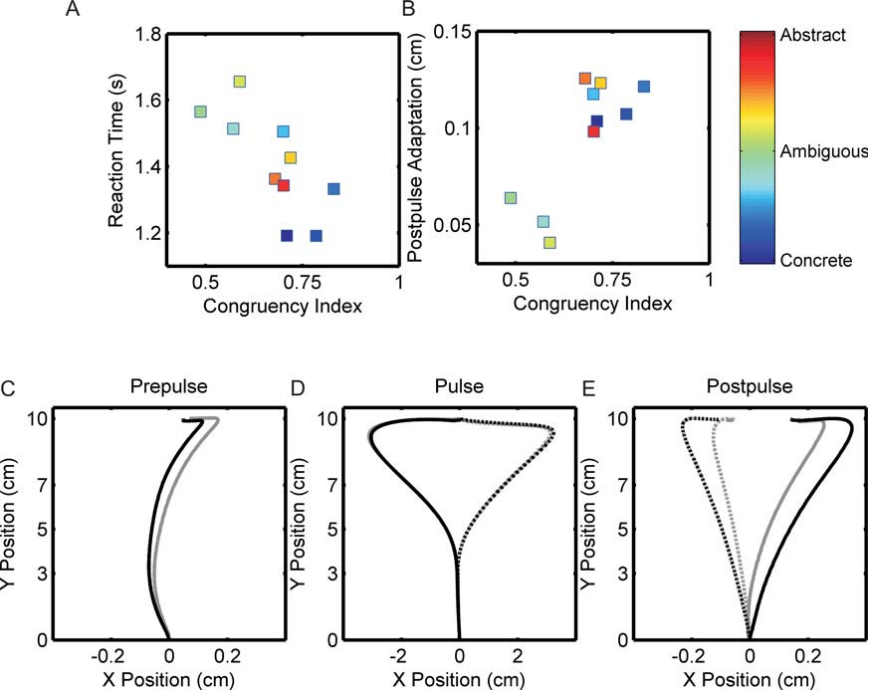
Decision-level uncertainty measured by comparisons of online to offline word categorization. The congruency index measured the similarity of word categorization from online to offline categorization across subjects. For each subject, words that maintained categorization were assigned a 1 and words that were switched were given a zero. Averaged across subjects, words were grouped into 10 equally sized bins dependent on congruency index. Colorbar (side) represents the linear spectrum of subjects' offline word survey of concreteness from most concrete (blue) to most abstract (red). A) The greater the congruency index (maintained categorizations), the faster the RT. These congruent words were words that best fell into either concrete or abstract categories during the offline word survey. B) The postpulse adaptation correlates with the congruency index. Subjects adapted more on congruent words (index closer to 1), than on incongruent words (closer to 0). C) Average movement trajectory preceding pulsed movements with either low congruency indices (gray), less than the mean congruency index, and movements with high congruency indices (black), greater than the mean congruency index. D) Average pulse movement trajectory for low (gray) and high (black) congruency index words for leftward (solid) and rightward (dashed) force perturbations. E) Average postpulse movement trajectory, minus average prepulse movement trajectory, following leftward (solid) and rightward (dashed) pulsed movements with low (gray) and high (black) congruency index words.

We generated a congruency index to quantify, across subjects, the categorization difficulty for each word. A congruency index closer to 1 indicates that subjects maintained the same categorization, while a congruency index closer to 0 indicates that subjects changed the categorization. The resultant congruency index spanned from 0.487 to 0.832 (figure 4). RTs were longer for words with lower congruency indices and RTs were correspondingly shorter for words with higher congruency indices (figure 4a, slope different from zero, r = −0.7323, p = 0.016). More strikingly, adaptation was significantly correlated with the congruency index, such that postpulse PD decreased with decreasing congruency indices (figure 4b, slope different from zero, r = 0.7839, p = 0.007). Postpulse PD was reduced by 50% for words with low congruency compared to words that had a high congruency.

A post-hoc analysis of the movement trajectories corresponding to low and high congruency indices further illustrated the decreased adaptation following difficult to categorize words. We separated the movements into two groups of congruency indices; one group with movements lower and one group with movements higher than the mean congruency index ( = 0.678). The average movement trajectory immediately preceding pulsed movements with low (<0.678) or high congruency (>0.678) were similar to each other (figure 4c). In addition, pulsed movement trajectories were nearly identical for low and high congruent word indices (figure 4d). However, the postpulse movement trajectory following low congruency index words showed substantially less adaptation than the postpulse movement following high congruency indices (figure 4e). Postpulse PD for low congruency index words was significantly less than the postpulse PD for high congruency index words (paired t-test, p = 0.004); PD for low congruency was 0.052±0.031 cm and for high congruency index words was 0.114±0.034 cm. This decreased adaptation was apparent throughout the entire movement.

### Comparison of adaptation following movements without word categorizations (single-task movements)

Participants also performed a single-task experiment, in which they did not perform the word categorization task. Postpulse movements in the single task showed significant adaptation. We computed the initial direction angle and the PD at 3, 5, and 7 cm. The adaptation in the single task was nearly the same as the adaptation for concrete and abstract words, but was significantly larger than the adaptation following ambiguous words. For the single task, the initial direction angle was 1.215±0.241°, which was larger than the angle for ambiguous words (angle = 0.913±0.327°; t-test p = 0.064), but not significantly larger than following concrete (1.335±0.372°, t-test p = 0.520) or abstract words (1.189±0.527°, t-test p = 0.942). The PD at 3, 5, and 7 cm were significantly larger in the single task than the PD at 3, 5, and 7 cm following ambiguous words (pairwise t-test, p = 0.0014), but were nearly equivalent for adaptation following concrete (pairwise t-test, p = 0.201) and abstract words (p = 0.927). For example, the PD at 5 cm was 0.100±0.022 cm in the single task, while it was 0.114±0.032 cm, 0.102±0.046 cm, and 0.079±0.029 cm, following concrete, abstract, and ambiguous words respectively.

The adaptation in the single task was nearly the same as the adaptation following concrete and abstract words, but was much larger than the adaptation following ambiguous words. These results suggest that performing the dual task itself does not cause a generalized decrement in adaptation, but rather when the dual task is difficult, in the case for ambiguous words, there is a specific interference effect. Our analysis of subject's congruency between online and offline word categorizations, suggests that it is not the word category itself that causes interference but rather the difficulty of categorization. When the PD at 5 cm in the postpulse movement of the single task is compared to the PD at 5 cm following low and high congruent words, we find that single-task adaptation and adaptation following high congruent words were very similar (PD = 0.114±0.03 t-test, p = 0.570). However, the adaptation on low congruent words (PD = 0.052±0.031) is over 50% less than the adaptation in the single task (t-test, p = 0.005). The similarity between single-task adaptation and adaptation following highly congruent words further supports the claim that easy to categorize words to do not burden decision-level process, however, hard to categorize words place a heavy burden on decision-level processing and we therefore see impairments in adaptation. These results suggest that simply performing the secondary task does not cause generalized interference with adaptation, but when the secondary task is difficult, then significant impairment in adaptation results.

## Discussion

The purpose of the concrete or abstract word categorization task was to vary the trial-to-trial decision-level difficulty of the task. Subjects were the slowest to categorize words that were ambiguous to either the concrete or the abstract category, suggesting that these trials were more difficult and required a greater degree of processing resources. Within the dual task, we observed a correlation between the decision-level difficulty of a particular word categorization on a pulsed movement and the subsequent motor adaptation on the next trial. Words ambiguous to the concrete or abstract categories were more difficult to categorize and lead to a marked decrement in speed of categorization and subsequently less postpulse adaptation. These results are not due to a generalized dual-task effect or context change because all of these results were observed within the dual task itself not between single- and dual-task experimental manipulations. Every word, regardless of its semantic category, required a button press for the categorization; therefore, the differential degradation in postpulse motor adaptation could not be due to motor planning interference of the button press. Thus, we suggest that the decision-level difficulty of the word categorization differentially burdened executive systems, relating to planning and decision-making, and consequently lead to interference in motor adaptation.

### Data-limited and Decision-limited Tasks

Previous experiments investigating the interaction between attention and motor control and learning have commonly employed data-limited secondary tasks [11], [17], [18], [25], [26] or the decision-limited secondary task was of unknown varying difficulty [15], [16]. A few experiments have attempted to vary the difficulty of the secondary task and measure the effect on motor control or adaptation [17], [26], however, these experiments utilized memory data-limited tasks [10], [27], [28]. Memory data-limited tasks generally involve the comparison between two stimuli; the difficulty of the task can be modulated by the similarity between the stimuli [10]. The performance limiting function in these tasks is the subjects' memory or representation of the input stimulus. In a study from our lab, we used a frequency discrimination task in which participants had to determine whether the second tone of two sequentially presented tones was either higher or lower in pitch than the first tone. By changing the size of the difference between the two tones we were able to manipulate participants' performance on the frequency discrimination task; however, this manipulation did not significantly affect feedback control or adaptation [17]. Adaptation was impaired by the frequency discrimination task, but we found that only the relative timing of tones presentation caused a specific interference effect. In another study, subjects performed treadmill walking while performing two levels of difficulty in a speeded reaction time task [26]; they found no effect of secondary task difficulty on walking. These results suggested that while the motor tasks shared resources with the secondary task the interference was not related to a capacity or resource limitation. The secondary tasks were data-limited and therefore difficulty manipulations may not have significantly burdened the executive system.

Here we find that motor adaptation is scaled when the secondary task difficulty is varied at the decision-level of processing. We suggest that utilizing decision-level tasks places more demands on attentional resources and therefore shows specific task difficulty interference. Since the movement task requires both visual and proprioceptive errors, we chose to divide attention by an auditory task. We utilized a semantic categorization task because it has been shown to vary subject RT depending on the meaning of the presented word [22]–[24]. In semantic categorization tasks, subjects are faster to categorize concrete words than abstract words [25], [26]. Thus, the concrete or abstract word categorization task provided a tool to vary the processing resource demands from trial-to-trial within a task. We observed longer reaction times when subjects categorized ambiguous words, which indicated that these words required longer mental processing and therefore induced a larger cognitive burden.

The trial-by-trial nature of the task paradigm allowed us to focus on the effect of dual-task interference on motor adaptation from one movement to the next movement. This allows us to precisely identify the direct effect of the decision-level processing burden of a secondary task on both within-movement feedback and across-movement adaptation. The formation of a new motor memory of novel forces may involve multiple processes working on multiple timescales [29]; our experimental design and trial-by-trial analysis of adaptation to occasional force pulses identifies the cognitive components of the fastest temporal processes of motor adaptation. Adaptation to repeatable and learnable forces may engage different neural systems than when forces are random and unlearnable [30]–[32].

### Categorization Uncertainty

To divorce analysis from semantic word properties, we investigated subject RT and postpulse PD on words in which subjects switched their response from during the dual task (online) to the word survey (offline). In post-hoc analysis, subjects had faster RTs when they maintained their categorization from online to offline. When subjects were certain of their decision they responded faster and maintained the same categorization from online to offline, while when subjects were uncertain they had slower RTs and changed online to offline categorization. We assessed this effect on a trial-by-trial basis by assigning a congruency index, a zero for switching categorization and a one for maintaining categorization, for each word. By averaging across subjects, we determined the decision uncertainty for each word independent of the semantic properties of the word. We found a strong correlation between word uncertainty and both RT and PD.

Why is there interference between semantic categorization uncertainty and motor adaptation? In the concrete or abstract task, ambiguous words created category uncertainty and response conflict. In situations of response conflict [5] or unexpected errors [33]–[35], it has been suggested that cognitive control is recruited to monitor or modify behavior. Monitoring behavioral performance in either cognitive tasks or motor tasks is critical to the successful performance and learning of the task [36]. We suggest that cognitive control is recruited to aid in categorization of ambiguous words and to aid in motor adaptation following unexpected movement errors. On pulsed trials, subjects experience an unexpected movement error. When pulsed movements coincide with ambiguous word categorizations cognitive control processing resources may be taxed by the conflict between the categories and the unexpected error. The simultaneous taxing of cognitive control slows word categorization and subsequently motor adaptation. The interference between these tasks suggests that they share common processing, and cognitive control may be the underlying process that subserves additional performance monitoring in the motor task.

Neuroanatomical studies have also suggested that similar neural systems are engaged by categorization tasks and during the early stages of a motor learning task. Prefrontal cortex [6] and anterior cingulate [37] activity has often been reported in categorization tasks, situations with errors [38] or response conflict [5], [39], [40], and during the early stages of motor learning [32], [41], [42]. These areas have been suggested to play roles in attention and working memory [6], [43], [44], performance monitoring [45], and response/action selection [46]. In addition, basal ganglia, specifically the cauduate nucleus, has shown involvement in both motor learning tasks and category learning tasks [47]–[49]. This neuroanatomical overlap further suggests a mechanism for general performance monitoring regardless of the nature of the task.

**Figure 5:**
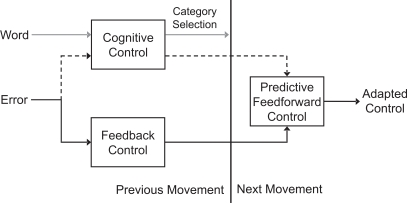
Possible model of systems involved in within-movement and across-movement motor control. During the pulsed movement, movement errors engage the feedback control system to correct the movement online (solid route) and the errors engage cognitive control systems (dashed route). The word categorization task also engages cognitive control differentially depending on the category ambiguity of the word (gray route). Feedback error learning systems update the predictive feedforward control on the next movement (solid route). Cognitive control also influences predictive control on the next movement (dashed route), but is degraded depending on the category ambiguity of the presented word.

The direct linkage between the functional recruitment of executive control for motor control and learning has not been well established. We hypothesize that the force-induced movement error in the pulsed trial is processed by two, possibly separate, control routes (figure 5). The external force perturbation induces proprioceptive and visual errors into the movement. Sensory signals of the perturbed movement engage the motor system's feedback controller (figure 5, solid route) and cognitive control (figure 5, dashed route).

The feedback controller corrects the movement online, and through feedback error learning [2], [50], updates predictive feedforward control on the subsequent movement. This model does not require an actual feedback correction to update predictive control on the next movement. Some ballistic movements, such as shooting a basketball, do not allow mid-movement correction based on these feedback processes. We nevertheless hypothesize that the automatized calculation of movement error that would have underlain mid-movement correction could be used to update control even if the correction, because of movement timing, cannot be expressed. In addition, the unexpected error engages cognitive control, which also evaluates the error and introduces an additional control signal or strategy to update predictive control on the next movement (figure 5, dashed route). During the dual task, the word categorization task (figure 5, gray route) strains the processing resources of cognitive control depending on the ambiguity of the word, which subsequently leads to degradation in the cognitive control signal.

We currently do not propose a motor adaptation route between cognitive control and within-movement feedback (figure 5). The lack of interference between the categorization task and within-movement feedback control suggests that cognitive control does not play a dominate role in the corrective feedback control of arm movements. In previous work from our lab, we also did not see any effect of the secondary task on the within movement feedback control following a force perturbation [17]. However, in both experiments subjects reaching movements are relatively short (<1 second), therefore, feedback control may be dominated more by reflexes than voluntary corrective control strategies. During long periods of wrist posture stabilization, both behavioral and neural correlates of changes in corrective control strategies on a moment-to-moment basis have been reported [51]. Combining a longer feedback control task with a secondary task may expose any present interaction between cognitive control and feedback control. In addition, under our current experimental paradigm we cannot determine if this lack of interference is bidirectional, such that cognitive control does not affect feedback control and feedback control does not interact with cognitive control of the error signal. A task with and without online feedback corrections could better investigate the bidirectional interaction between cognitive control and feedback control.

Two separate control routes for motor adaptation have been suggested by several studies [52], [53]. In visuomotor rotations, when the rotation is introduced abruptly subjects become aware of the rotation and reduce the error quickly. When the rotation is introduced gradually, subjects still learn the rotation, but without awareness of the rotation. When the rotation is turned off, subjects show stronger aftereffects in the gradual condition than in the abrupt condition suggesting that learning may be mediated by two different systems [54]. In addition, when subjects were given explicit knowledge of a visuomotor rotation they were able to reduce the error initially, but towards the end of training subjects made increasingly large errors [55]. This suggests that explicit control strategies could not replace sensorimotor adaptation and were eventually overridden by an adaptive motor system [55]. An earlier study of prism adaptation also provided anecdotal evidence that an explicit control strategy could reduce errors initially, but was eventually overridden by underlying visuomotor recalibration [56]. These results suggest that sensorimotor adaptation is influenced by both unconscious processes and by cognitive strategies (figure 5).

The pulsatile forces occur infrequently, so we do not suggest here that subjects use either explicit or implicit strategies to predict these forces. We instead suggest that the perturbations induce cognitive control in the transformation of movement sensation into incremental adaptation, and that cognitive load interferes with this transformation. The precise contribution of cognitive control may in some cases drive participants to utilize an explicit strategy to improve performance while in other cases it may just aid in learning by adding another learning signal on top of an automatic (or implicit) motor learning process without the participants developing an explicit strategy. There are many cognitive tasks, which are thought to engage the executive system, but have implicit or automatic consequences. During a flanker task, when a participant commits an error in a previous trial, the participant is slower on the next trial and shows improved performance. This Gratton effect [25], suggests that participants are implementing a controlled response following an error to ensure accuracy. This trial-by-trial control of behavior based upon previous performance is not necessarily explicit, but is thought to engage systems underlying classically-defined cognitive processes [34]. The executive system may play a similar functional role during motor learning.

## Materials and Methods

### Experiment Design

Fourteen healthy right-handed human subjects (5 female and 9 male), aged 21–30 years, participated in the two-day experiment. The Washington University Hilltop Human Studies Committee approved the experimental protocol and all subjects gave their informed consent. On the first day of the experiment, subjects made horizontal reaching movements while holding a manipulandum. The first day of the experiment was designed to allow the subjects to learn the passive dynamics of the manipulandum and the basics of the movement task. On the second day of the experiment, subjects performed the dual-task experiment, which was comprised of a movement task and a concrete/abstract decision task (figure 1a). Subjects performed 360 horizontal reaching movements. In 90 of these movements, we generated the transient force perturbations. In 150 of the 360 movements, we presented a word to the subjects, via headphones, and subjects were instructed to categorize the word as being a concrete or an abstract word.

### Word Selection

To determine the words for the concrete or abstract categorization task, we initially gathered words from The English Lexicon Project [57]. The words were selected based upon the following criterion: words with 1 syllable, a log of the hyperspace analog of language (HAL) frequency from 5 to 10 [58], and a lexical decision reaction time of less than 600 ms. From this criterion, 400 words were selected for additional screening.

A separate group of 8 subjects (3 female and 5 male) scored each of the 400 words on a scale from 1 to 5: 1 being concrete, 2 being somewhat concrete, 3 being neither completely concrete nor abstract, 4 being somewhat abstract, and 5 being abstract. The subjects' scores were compiled and the words were binned into three categories: concrete (scores of 1 to 2), ambiguous (score of 3), abstract (scores of 4 and 5). Fifty words with the lowest variance were selected from each category. Based upon the MRC Psycholingusitic Database [59], the concrete, abstract, and ambiguous words had concreteness ratings of 605, 351, and 464, respectively. These 50 concrete, 50 abstract, and 50 ambiguous words were used in the concrete or abstract task.

Following the movement task practice (see below) on the first day, the 150 words were randomly presented to each subject, through headphones. Subjects were asked to repeat each word to the experimenter to ensure that the subject could correctly identify the word. If the word was incorrectly identified, the word was clarified by the experimenter and the sound file was repeated. Subjects were naïve to the concrete or abstract task.

The 150 words were converted into audio files using text-to-speech software (Wizzard Software, Pittsburgh, PA). To make all of the audio files the same presentation duration without changing the pitch of the audio file, pitch cycles were added or deleted automatically to lengthen or shorten each sound file to 500 ms using Adobe Audition software (Adobe, San Jose, CA). All audio files were encoded to 16 bits at 16 kHz in Matlab (Mathworks, Natick, MA), and transformed into audio signals by a soundcard (Creative SoundBlaster, Milpitas, CA), and played through headphones (Koss UR29, Milwaukee, WI). Subjects were allowed to adjust the volume of the headphones to a comfortable volume.

### Movement Practice Task (Day 1)

The first day of the experiment was designed to allow the subjects to become accustomed to the task and the passive dynamics of the robotic manipulandum. Subjects made 4 sets of 180 movements while holding a five link, two bar robotic manipulandum (Interactive Motion Technologies, Cambridge, MA) with their right-hand (dominant hand). Subjects handedness was determined by the Edinburgh handedness inventory [60]; all subjects were right-hand dominant. Movements were 10 cm in length and were directed away from the body in the horizontal plane. Subjects were instructed to move their hand from an initial starting position to a single visually displayed target and come to a complete stop within the target. An LCD monitor displayed the visual target and cursor positions. If the subject reached the target within 450–550 ms, then the target turned green and burst. However, if the subject was too slow or too fast, then the target turned blue and red, respectively. After the subject reached the target, the target was removed and the robotic manipulandum returned the subjects' hand to the start position.

The manipulandum moved in the horizontal plane by revolution at two joints. Subject hand position and velocity were recorded by encoders on the robotic manipulandum. The manipulandum estimated states and generated forces at 1000 Hz. The manipulandum was capable of generating dynamic forces through two-brushless DC motors, but during the movement training-task, no forces were generated during the movement.

### Dual task (Day 2)

On the second day of the experiment, subjects made 4 sets of 180 movements while holding the manipulandum. The movement task was nearly identical to the movement training task (day 1) except that on 25% of the movements, subjects experienced transient viscous force perturbation midway through the movement (figure 1a). The force in the x-direction (perpendicular to the target direction) depended on the hand position in the y-direction (toward the target direction). The forces were centered at a y-displacement of 5 cm with a width of 2 cm (Equation 1).
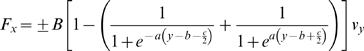
(1)where F_x_ is the forces in x-direction, B ( = 40 Nm^−1^ s) is the viscous gain of the force, a ( = 3.33 cm^−1^) controls the shape of the force and it was chosen to generate an approximate Gaussian shape, b ( = 5 cm) is the center of the pulse, c ( = 2 cm) sets the width of the pulse, y is the distance toward the target, v_y_ is the y-velocity. The forces were presented pseudorandomly such that perturbations never occurred in succession. In addition, the force direction was balanced leftward and rightward such that no lasting motor memory could develop.

On 2 of the 4 sets of movements, subjects performed the movement task with the force perturbations but without the categorization task (single task). On the other 2 sets, subjects performed the word categorization task (dual task) while performing the movements identically to the single task. In the dual task, subjects made concrete or abstract judgments on words presented, via headphones, on 150 of the 360 movements. The word was presented when the hand reached 5 cm in the y-direction (figure 1a). Subjects were instructed to decide if the presented word was more concrete or more abstract by pressing a corresponding button on a 3-button mouse (Logitech, Fremont, CA). Subjects responded with either their left index or left ring finger. Subjects were instructed to categorize each word to the best of their ability as quickly as possible. Subjects' reaction times were quantified as the time interval between the start of the word audio file and the subjects' mouse button press. The words were presented pseudorandomly on movements immediately before a perturbation (prepulse; 15 out of 90 movements had words), movements with a perturbation (pulse; 60 out of 90), following a perturbation (postpulse; 60 out of 90), and other movements (15 out of 90). On movements in which no word was presented, subjects pressed the middle mouse button with their left-hand middle finger. Subjects could not go onto the next movement without making a decision. No feedback was provided since there is not always a correct or incorrect categorization for a particular word.

Following the dual task on day 2, subjects were provided with a survey of the 150 words they heard in the dual-task experiment. Subjects were asked to score each of the words from 1 to 5, with 1 being the most concrete and 5 being the most abstract. This survey provided a means to compare subject's divided attention categorization during the dual task (online) and their undivided attention categorization (offline) for each of the 150 words.

### Data Analysis

Subject hand kinematics and word categorization task responses were analyzed in Matlab (Mathworks, Natick, MA). All position data were shifted such that all movements started at the same position (x = 0 and y = 0). We used a 4^th^ order Savitsky-Golay filter to determine acceleration from 25 ms windows of velocity data. Data for individual subjects were averaged and the means within a subject were compared across subjects. To combine pulsed movements across leftward and rightward pulse directions, we subtracted leftward from rightward movements and divided by 2. On postpulse movements, adaptation is in the opposite direction of the force pulse in the pulsed movement; leftward pulses cause adaptation in the positive x-direction and rightward pulses cause adaptation in the negative x-direction. Therefore, for postpulse movements, we combined across pulse direction by subtracting rightward from leftward metrics and dividing by two. Analysis of variance (ANOVA) was used to compare differences across all word categories, while paired t-tests were used to compare differences between word categories. All metrics are reported as mean +/− the 95% confidence interval of the mean.

To better preserve temporal differences in the feedback response, we aligned all movements by the time of maximum force before averaging across movements. We chose metrics used to quantify the early and late stages of feedback control during the pulsed movement. Maximum perpendicular displacement (PD) and time of corrective control initiation (Tc) were used to quantify the short-loop feedback response. Settling time and integral squared error (ISE) were used to quantify the long-loop feedback response. Corrective control initiation was defined as the time from the maximum force until the x-component of acceleration of the hand changed. Settling time was measured from the time of maximum force until the hand reached 10% of its final x-position. ISE was the time-integral of the square of the x-component of hand position measured from the time of corrective control initiation.

In postpulse movements, to better preserve differences in adaptation early into movement we aligned all movements by the time of movement initiation. To quantify adaptation in postpulse movements, three analyses were used to define the kinematic features of adaptation: initial movement direction, PD at 3, 5, and 7 cm, and total movement area. Initial movement direction was used to determine adaptation early into movement. To compute initial movement direction, a line was drawn between the starting position and the position of the hand at peak speed; the angle between this line and the straight-line between the starting position and the target determined the initial movement direction [61]. Each subject's mean PD at 3, 5, and 7 cm into movement was computed for postpulse movements following leftward and rightward perturbations to quantify adaptation midway into movement. Total movement area was used to quantify adaptation during the entire movement. The area was defined as the sum of the positive and negative area of the x-component of hand position. This area metric was also used to quantify prepulse movement curvature. These metrics of adaptation were quantified by subtracting the subjects' average prepulse metrics from the subjects' postpulse movement metrics.

We analyzed the semantic categorization RT and postpulse PD according to an individual subject's concreteness rating for each word during the offline word categorization. The rating for each word that occurred on a pulsed trial was averaged across subjects and resultant ratings were sorted and grouped into 10 equally sized bins, and then averaged within the bin. The word RTs on pulsed movements and the following postpulse PDs were grouped into the 10 word group bins, and then averaged together.

The congruency index provides an estimate of the subjects' word categorization uncertainty. A particular word was given a congruency of 1 if the subject maintained the same categorization from online during the dual task to the offline survey rating for that particular word. However, if the subject changed the categorization from online to offline, then this word was given a congruency index of 0. We averaged the congruency index for every word across subjects, sorted the indices, binned the indices into 10 equally sized bins, and averaged RTs and PDs within the bin.

### Statistical Tests

Whenever we investigated the pairwise differences between two conditions, even when those two conditions were a subset of more than three, we used standard uncorrected t-tests to have the most sensitivity in avoiding Type II statistical errors. When these uncorrected t-tests revealed significant differences, disproving null hypotheses, we accounted for possible Type I statistical errors by applying Bonferroni corrections. These corrections resulted in p-values that retained significant results (one corrected p = 0.03; all others p<0.01).
